# Circadian rhythms and pancreas physiology: A review

**DOI:** 10.3389/fendo.2022.920261

**Published:** 2022-08-10

**Authors:** Karl Chan, F. Susan Wong, James Alexander Pearson

**Affiliations:** ^1^ Diabetes Research Group, Division of Infection and Immunity, School of Medicine, Cardiff University, Cardiff, United Kingdom; ^2^ Systems Immunity Research Institute, School of Medicine, Cardiff University, Cardiff, United Kingdom

**Keywords:** circadian rhythm, pancreas, insulin, metabolism, obesity

## Abstract

Type 2 diabetes mellitus, obesity and metabolic syndrome are becoming more prevalent worldwide and will present an increasingly challenging burden on healthcare systems. These interlinked metabolic abnormalities predispose affected individuals to a plethora of complications and comorbidities. Furthermore, diabetes is estimated by the World Health Organization to have caused 1.5 million deaths in 2019, with this figure projected to rise in coming years. This highlights the need for further research into the management of metabolic diseases and their complications. Studies on circadian rhythms, referring to physiological and behavioral changes which repeat approximately every 24 hours, may provide important insight into managing metabolic disease. Epidemiological studies show that populations who are at risk of circadian disruption such as night shift workers and regular long-haul flyers are also at an elevated risk of metabolic abnormalities such as insulin resistance and obesity. Aberrant expression of circadian genes appears to contribute to the dysregulation of metabolic functions such as insulin secretion, glucose homeostasis and energy expenditure. The potential clinical implications of these findings have been highlighted in animal studies and pilot studies in humans giving rise to the development of circadian interventions strategies including chronotherapy (time-specific therapy), time-restricted feeding, and circadian molecule stabilizers/analogues. Research into these areas will provide insights into the future of circadian medicine in metabolic diseases. In this review, we discuss the physiology of metabolism and the role of circadian timing in regulating these metabolic functions. Also, we review the clinical aspects of circadian physiology and the impact that ongoing and future research may have on the management of metabolic disease.

## Introduction

**Figure 1:**
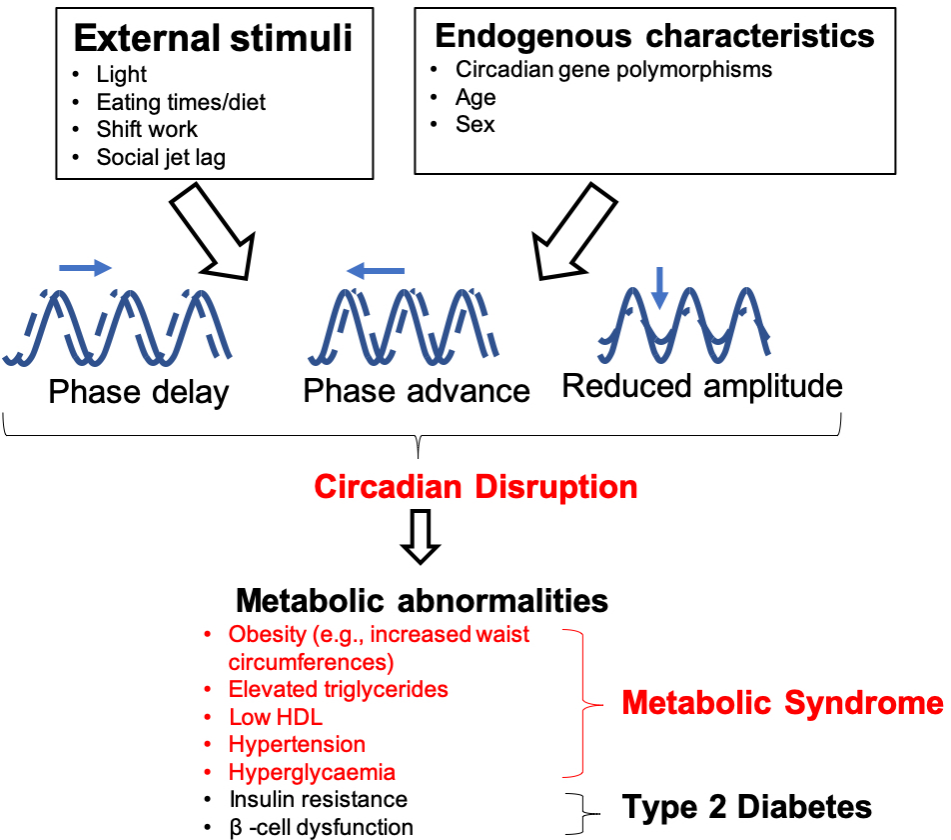
Influences on, and consequences of, circadian rhythm disruption. Both endogenous and external factors can predispose individuals to circadian disruption. This can cause dysfunction of peripheral oscillators, which are involved in the regulation of metabolic functions such as body weight homeostasis, glucose metabolism and β-cell function. Individuals who experience circadian disruption, which may be through phase delays/advances or by changes in amplitude (difference between peak and trough of the rhythms) are at an elevated risk of developing metabolic abnormalities, which can lead to metabolic syndrome and T2DM.

Diabetes mellitus is estimated to affect 415 million adults worldwide, approximately 90% of whom have type 2 diabetes (1). Diabetes can lead to microvascular (e.g. retinopathy, nephropathy and neuropathy) and macrovascular complications which substantially increase the risk of developing cardiovascular disease and can substantially decrease the quality of life of these individuals (2). Managing diabetes and the associated complications will introduce more strain on healthcare systems worldwide as this disease becomes more prevalent (3). This highlights the need for both determining the importance of risk factors, which may modulate susceptibility to disease, and management of the metabolic syndrome (referring to metabolic abnormalities listed in **Figure 1**, which together increase the risk of cardiovascular diseases and type 2 diabetes), obesity and type 2 diabetes (T2DM).

Many physiological processes, including energy expenditure and glucose homeostasis are regulated by circadian rhythms (4, 5), which are 24-hour daily cycles of physiological and behavioral patterns (6). The circadian rhythm apparatus consists of a central master clock located in the suprachiasmatic nuclei (SCN) within the hypothalamus, which synchronizes peripheral oscillators located in various tissues such as the liver, pancreas, adipose tissue and skeletal muscle. The SCN is entrained by light (7, 8), which allows synchronization with the external environment i.e. the 24-hour light-dark cycle governed by the Earth’s rotation. This directs the central and peripheral clocks to adapt to changes in light, optimizing physiological processes to these daily cycles. Through a number of regulatory mechanisms (e.g. endocrine, neurological, thermal), the SCN coordinates responses with the peripheral clocks, which have their own phases, to ensure synchronized daily rhythms are maintained (9). In addition, peripheral rhythms can also be modulated, for example, by nutrient sensing (i.e. from food intake), hormonal cues and temperature. Although the SCN acts as the master pacemaker in the human body, it is evident that circadian oscillations are observable in almost every cell of the body and these rhythms may persist in isolation from the SCN (10).

Disruption of circadian rhythms exacerbates metabolic diseases that include T2DM, obesity and metabolic syndrome in both animal models and humans (11–15) (**Figure 1**). Experimental and epidemiological studies show that night shift-workers are more likely to develop metabolic abnormalities, predisposing these individuals to developing T2DM compared to daytime workers (16–22). This elevated risk of developing T2DM is also seen in populations with social jetlag, a condition characterized by disruption to an individual’s sleeping pattern, and thus circadian rhythms, due to social engagements, leading to individuals feeling “jet lagged” or tired (20, 21, 23).

**Figure 2:**
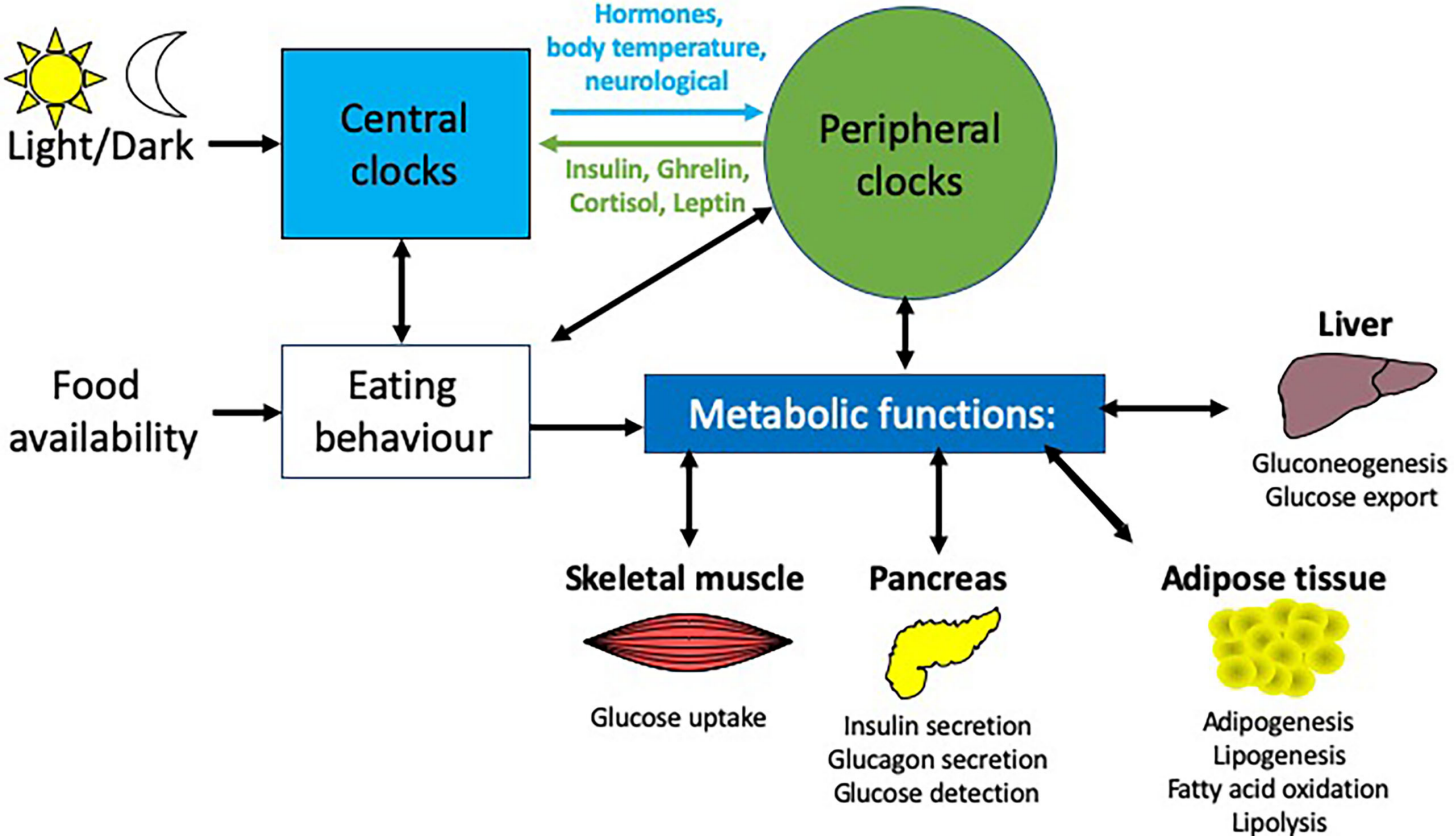
Circadian influences on different metabolic tissues. Light is the main entrainment factor for the SCN, the master pacemaker of the circadian system, which, through a number of signals e,g. hormones and neurotransmitters, synchronizes the circadian rhythms of peripheral tissues to light exposure. Crosstalk between these peripheral tissues and the brain enable feedback to modulate these rhythms e.g.the hormones insulin, ghrelin, leptin and cortisol provide feedback to the arcuate nucleus in the brain. There are many peripheral tissues, which regulate metabolic functions, including the liver, pancreas, skeletal muscle and adipose tissue, each of which exhibit their own rhythmicity. Together, these peripheral rhythms regulate many metabolic functions, including glucose homeostasis, insulin secretion and fatty acid metabolism.

**Figure 3:**
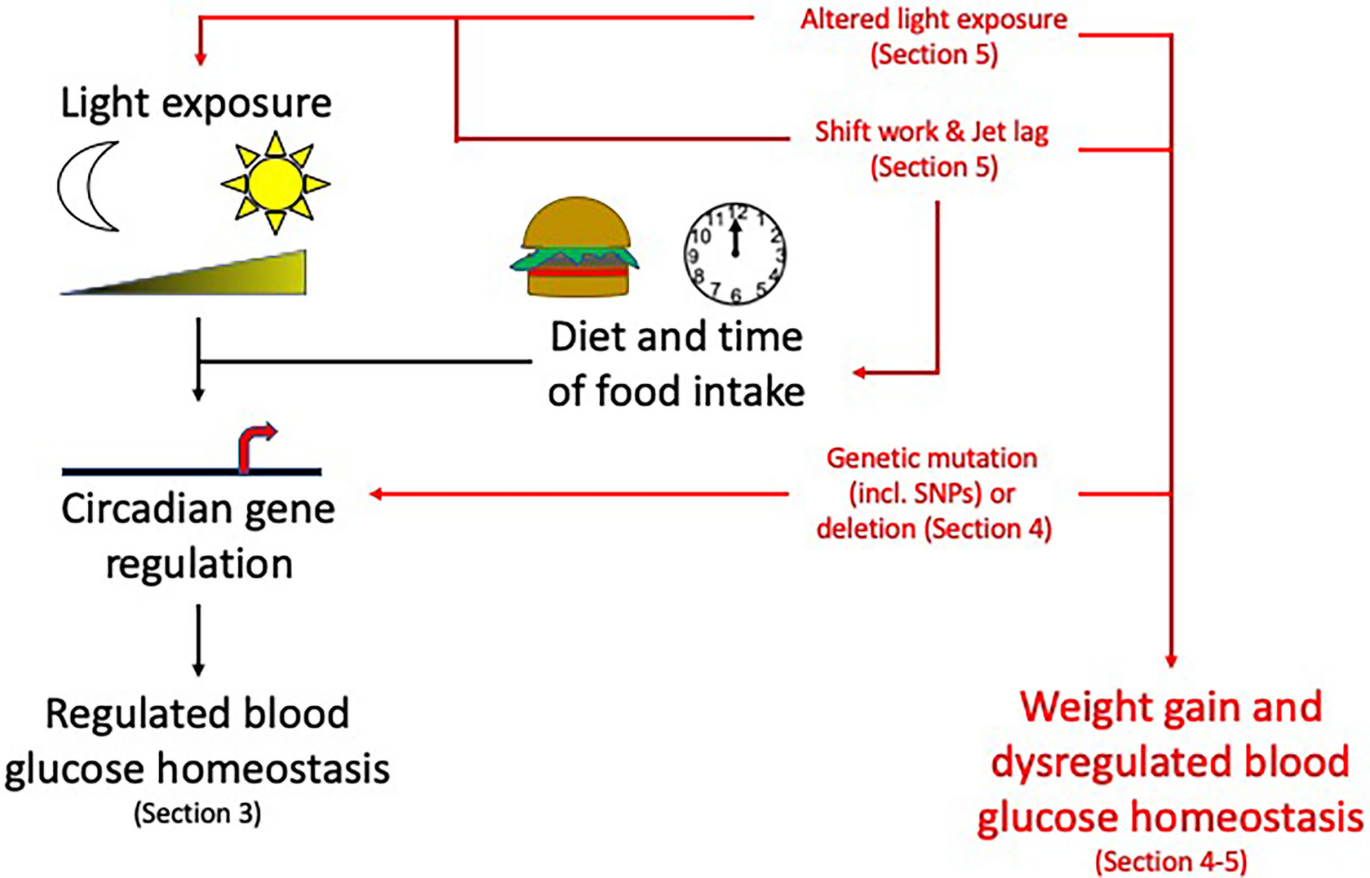
Circadian rhythms and their disruption: Topics to be discussed in this review. Circadian rhythms, influenced by light/dark cycles and diet and time of food intake, control many pancreas functions. Disruption to these circadian influences (altered light exposure, shift work/jet lag, genetic mutations) can lead to weight gain and dysregulated blood glucose homeostasis increasing the risk of developing obesity and T2DM. For information related to these factors altering the circadian rhythms please see the specific sections mentioned above within the review. Section 3: Circadian rhythms in the pancreas, Section 4: Molecular circadian rhythms in the pancreas and Section 5: Modulation of circadian rhythm.

Many central and peripheral hormones that influence metabolism exhibit circadian rhythmicity. This review will focus on the current understanding of how circadian rhythms can influence pancreatic physiology and the consequent effects on metabolism and how this knowledge may be used to enhance clinical management of T2DM, obesity and metabolic syndrome. While we will focus on the pancreas in this review, other metabolic tissues e.g. liver, skeletal muscle and adipose tissue are also altered by circadian rhythms (**Figure 2**). While providing background information on blood glucose homeostasis and insulin resistance to those less familiar with these concepts Section 2: (Pancreatic physiology), we will also highlight key ways circadian rhythms are disrupted and how they interact to increase the risk of obesity and T2DM (**Figure 3**) discussed in Section 3 (Circadian rhythms in the pancreas), 4 (Molecular circadian rhythms in the pancreas) and 5 (Modulation of circadian rhythm).

## Pancreatic physiology

The pancreas is a multifunctional organ which regulates metabolism and digestion through several endocrine and exocrine mechanisms (24). These tightly regulated, interrelated mechanisms are necessary for blood glucose homeostasis, lipolysis, and food intake (4, 5). Histologically, the pancreas contains clusters of exocrine cells known as acini which surround a network of interconnected ducts (25). The acini secrete inactive forms of pancreatic enzymes known as zymogens which subsequently enter the gut and become active digestive enzymes, including lipase, amylase and proteases. The endocrine cells of the pancreas are arranged in clusters known as the islets of Langerhans which contain α, β, γ and δ cells (26). Each of these cell populations within the islets secrete different hormones, as discussed later. Together, these pancreatic hormones regulate blood glucose homeostasis, food intake and insulin responses and are therefore integral to understanding T2DM and metabolic syndrome.

### Introduction to blood glucose homeostasis

**Figure 4:**
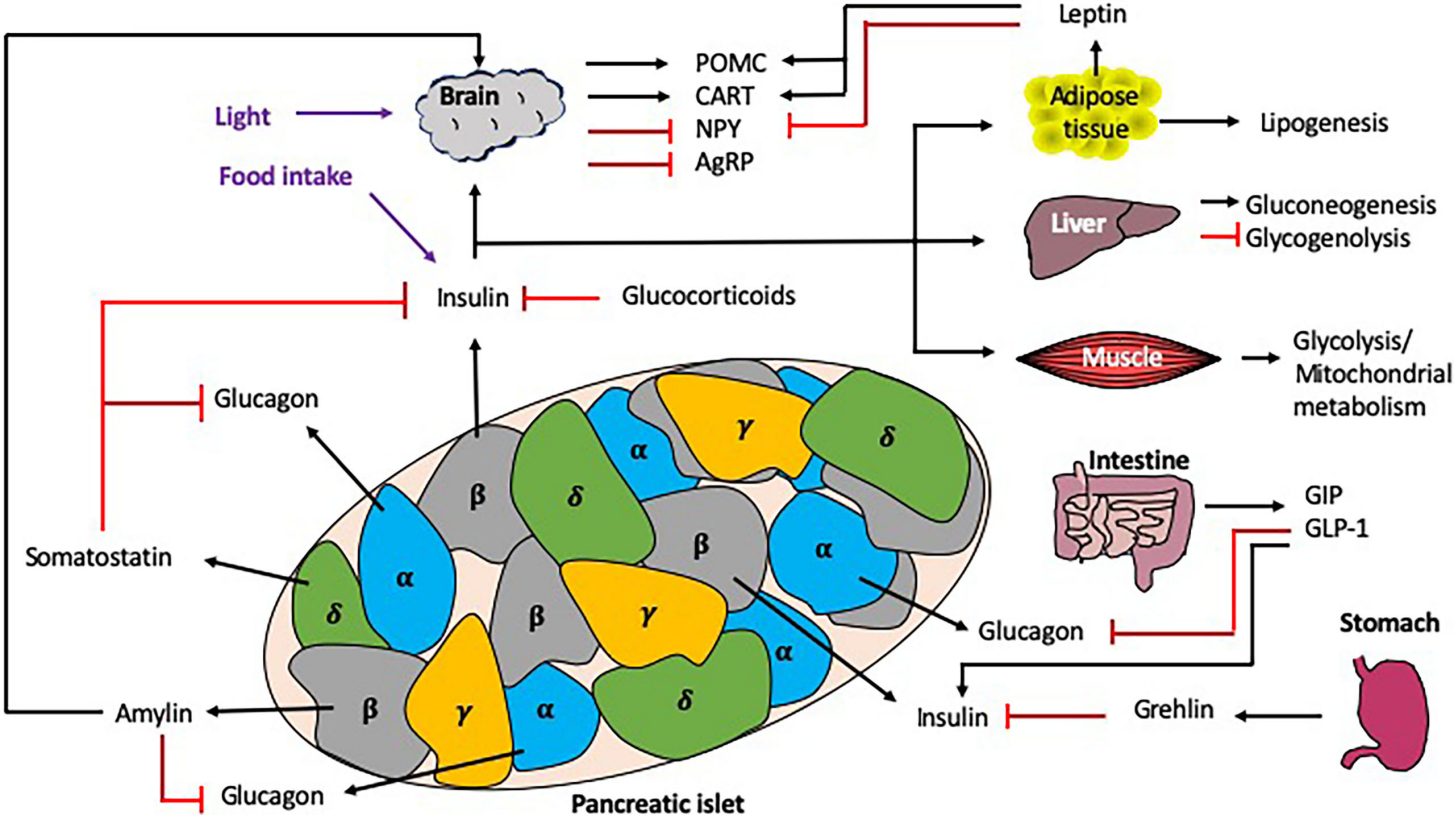
Interactions regulating blood glucose homeostasis. Blood glucose homeostasis is controlled by many signals both locally within the pancreas, and more distally, from the brain, liver, muscle, intestine, stomach and adipose tissue. Within the pancreatic islets, the α cells secrete glucagon, the β cells secrete insulin (and amylin), γcells secrete pancreatic polypeptide and the δ cells secrete somatostatin. In response to high glucose e.g. from dietary intake, the islet ββcells secrete insulin, which is detected by multiple tissues in the periphery, leading to the synthesis or induction of many molecules/pathways e.g. lipogenesis and gluconeogenesis, as well as the inhibition of others e.g. glycogenolysis. Importantly, there are many mechanisms of regulation to control the secretion of insulin both locally (e.g. glucagon and somatostatin) and more distally, e.g. intestine/stomach *via* hormones (e.g. grehlin and glucocorticoids) and incretins (GIP/GLP-1). Exogenous circadian modulation factors such as light and food intake (shown in purple) can also regulate blood glucose homeostasis, as can endogenous factors such as glucocorticoids, which also show rhythmicity. Black arrows indicate induction, red lines indicate inhibition. .

Blood glucose homeostasis is a highly regulated process, strongly influenced by both local (i.e. pancreas) and distal (i.e. liver, intestine, brain) signals (**Figure 4**). In this section, we discuss the role different hormones have on regulating blood glucose.

In normal pancreatic physiology, the β cells of the islets of Langerhans express glucose transporter type-2 (GLUT-2) molecules which detect changes in blood glucose levels, having high capacity and low affinity for glucose (27). Glucose enters the β cells through these high-capacity transporters and subsequently enters the glycolytic pathway and mitochondrial metabolism, which increases the cytoplasmic concentration of ATP leading to the gating of ATP-sensitive potassium channels (K_ATP_). The subsequent plasma membrane depolarization opens voltage-dependent calcium channels (VDCC) and allows an influx of calcium into the cell. The increased intracellular concentration of calcium causes the fusion of insulin granules with the cell membrane and the secretion of insulin.

Insulin exerts its effect on cells by binding to insulin receptors on the cell surface. These insulin receptors are homodimers, consisting of two α and two β subunits. Insulin binds to the extracellular α subunits, leading to autophosphorylation of the β subunits that are tyrosine receptor kinases (RTKs). These RTKs phosphorylate insulin receptor substrate (IRS) that activates downstream pathways mediating the cellular effects (28). Insulin acts on several body tissues including the liver, adipose tissue and skeletal muscle to allow the entry of glucose into these cells to undergo glycolysis and mitochondrial metabolism or anabolic processes such as glycogenesis or lipogenesis (29). Importantly, insulin inhibits hepatic gluconeogenesis and glucose secretion, regulating blood glucose levels. The liver is also sensitive to decreased blood concentrations of insulin, and this stimulates glucose synthesis and secretion. Insulin receptors are expressed throughout different regions of the brain (30). In the arcuate nucleus of the hypothalamus (ARC), insulin modulates anorexigenic and orexigenic neuronal activity. Anorexigenic neurons (pro-opiomelanocortin (POMC) and cocaine–amphetamine-regulated-transcript (CART)) are stimulated by insulin whilst orexigenic neurons [neuropeptide Y (NPY) and agouti-related peptide (AgRP)] are inhibited (31). These actions simultaneously decrease food intake and increase energy expenditure. Leptin, a hormone released by adipose cells and also controlled in a circadian manner (32), exerts a similar effect by stimulating POMC and CART, whilst inhibiting NPY neurons (33). Together leptin and insulin act as signals of adiposity which allow the body to regulate adipose tissue mass (34).

Glucagon, another pancreatic hormone, is secreted by α cells in the pancreatic islets in response to decreased blood glucose levels (35). It opposes the action of insulin in glucose control by stimulating glucose synthesis and secretion. Glucagon also stimulates ketogenesis and lipolysis in the liver. Glucagon levels in the hepatic portal vein are detected by the liver and this signal is relayed centrally *via* the vagal afferents to the ARC to reduce meal sizes by stimulating postprandial satiety (36). Additionally, glucagon is able to cross the blood-brain barrier (BBB) and has been shown to activate GPCR pathways in the ARC in animal models (37). This implies that glucagon may have a direct effect on the CNS to regulate food intake.

The hormone amylin is co-secreted with insulin by the pancreatic β cells, reducing food intake by inhibiting orexigenic neurons in the ARC (38). Amylin also activates the area postrema (AP) in the medulla oblongata of the brainstem to slow gastric emptying, inhibit gastrointestinal secretions and inhibit the postprandial secretion of glucagon (39).

Somatostatin, a hormone secreted by pancreatic δ cells, regulates digestion, food intake and glucose metabolism through endocrine, exocrine and neurological mechanisms. This hormone inhibits the secretion of the insulin and glucagon as well as prolactin, thyroid stimulating hormone, gastrin and secretin. In the gut, somatostatin inhibits digestive secretions including pancreatic enzymes, gastric acid and bile.

Corticosteroids, produced in the adrenals, regulate a variety of physiological processes including stress responses, immune responses and inflammation, blood glucose homeostasis and electrolyte balance (40). The secretion of these hormones is also regulated by the circadian clock and follows a 24-hour cycle (41, 42). The two main classes of corticosteroids are mineralocorticoids and glucocorticoids (43). Whereas mineralocorticoids, such as aldosterone, regulate fluid and electrolyte balances by modulating the activity of the renal tubules (44), glucocorticoids have anti-inflammatory effects and also regulate carbohydrate, protein and lipid metabolism (45). Cortisol is the main endogenous glucocorticoid in human physiology and is also released as part of the stress response, which in the presence of hypoglycemia, increases blood glucose levels, in both stress and hypoglycaemia, by stimulating gluconeogenesis (46).

Incretins are peptide hormones that are secreted by gut cells postprandially to regulate blood glucose levels and nutrient absorption (47). The two main incretins are glucagon-like peptide-1 (GLP-1) and gastric inhibitory peptide (GIP) (48, 49), and they decrease blood glucose levels by facilitating the secretion of insulin from pancreatic β cells (50). GLP-1 also inhibits the secretion of glucagon by pancreatic α cells. In addition, incretins also slow the rate of gastric emptying to regulate the rate of nutrient absorption (51). Both GLP-1 and GIP are inactivated by dipeptidyl peptidase-4 (DPP-4) (47). Several GLP-1 analogues and DPP-4 inhibitors are used clinically in the management of T2DM (52). Preliminary reports indicate that GIP analogues may also be effective in the management of T2DM, although further investigation is needed to elucidate the clinical efficacy of these drugs (53–56).

Ghrelin initiates appetite by stimulating orexigenic NPY neurons and inhibiting POMC neurons in the ARC, and is also secreted by gastrointestinal cells, located predominantly in the stomach. Ghrelin also raises blood glucose levels by inhibiting glucose-stimulated insulin secretion (GSIS) and impairing glucose tolerance (57, 58).

### An introduction to insulin resistance

**Figure 5:**
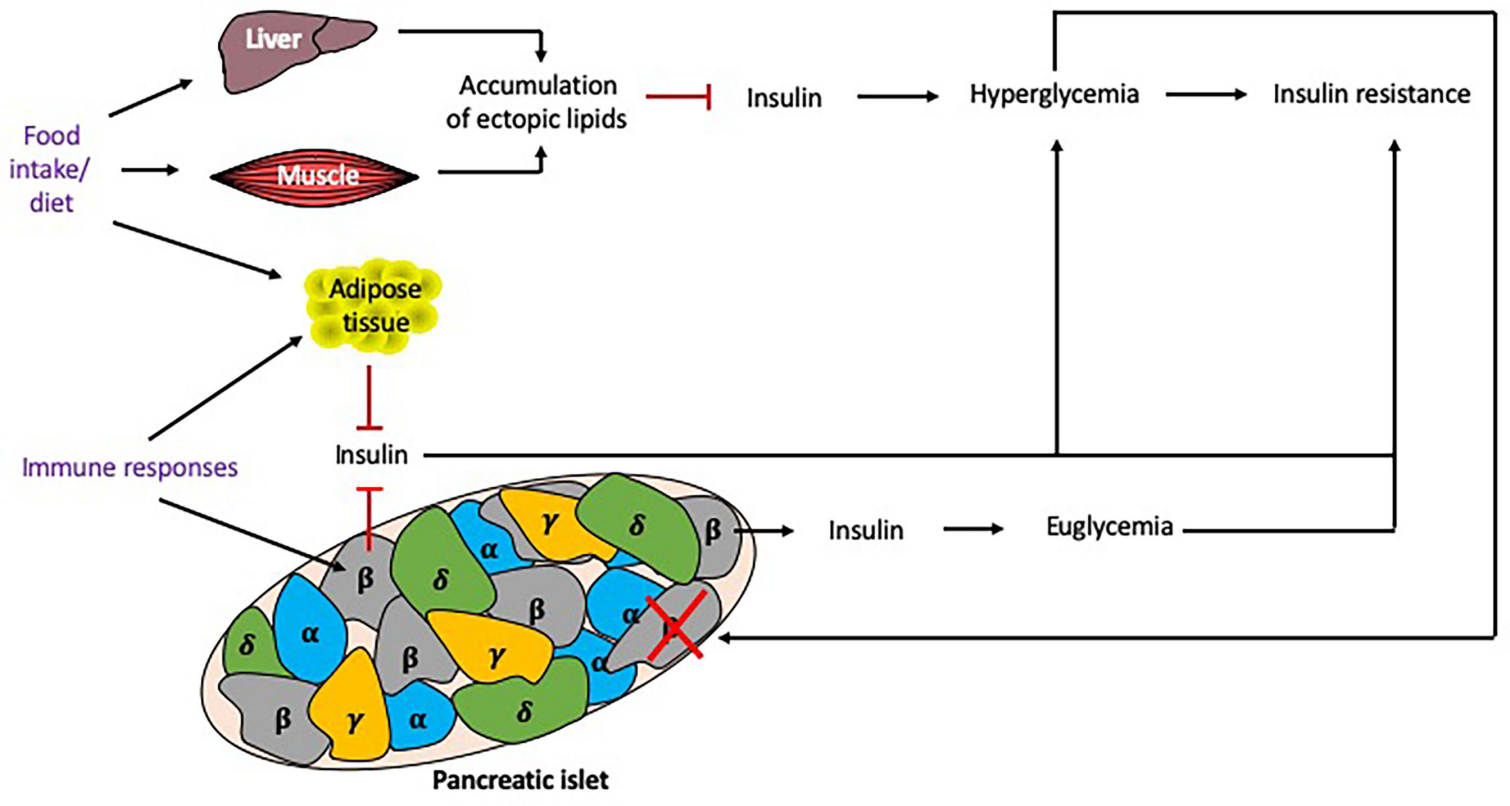
Factors influencing insulin resistance. Multiple factors may contribute to the development of insulin resistance. Excess food intake and the type of diet eaten can promote the accumulation of lipids in tissues, reducing insulin signaling and causing hyperglycemia and insulin resistance. Chronic hyperglycemia induces β cell stress, leading to β cells that fail to secrete sufficient insulin to maintain euglycemia, or exhausted/destroyed β cells, which do not secrete insulin, further promoting hyperglycemia. Genetics play an important role as some individuals may develop insulin resistance but remain euglycemic. Immune responses, particularly cytokines secreted from macrophages, can also promote adiposity and insulin resistance. Responses to food intake and immune responses (shown in purple) can be altered by the time of day and thus circadian rhythms may alter these responses. Black arrows indicate induction, red lines indicate inhibition.

Insulin resistance, a core component in the pathophysiology of T2DM, is associated with the metabolic syndrome (MS) and obesity (59), and influenced by many factors (**Figure 5**). Insulin resistance occurs in the presence of chronic energy excess, which leads to accumulation of ectopic lipids in hepatic and skeletal muscle tissue, impairing insulin signaling in these tissues, resulting in hyperglycemia. Although insulin resistance and obesity are strong risk factors for T2DM, these factors alone are not sufficient to produce hyperglycemia (60). β cell dysfunction in the islets of Langerhans is also required to produce T2DM, although the degree of β cell function and insulin resistance varies between individuals. β cell dysfunction results from an inability to detect elevated glucose levels to stimulate an appropriate secretion of insulin (59), which exacerbates hyperglycemia.

The mechanisms of β cell dysfunction are not fully understood. A number of factors are believed to contribute to this phenomenon, including proinflammatory cytokines which are associated with obesity and induce mitochondrial stress in β cells (61). Macrophage infiltration into adipose tissue is considered to be the main source of cytokines in obese individuals, with both macrophage infiltration into the adipose tissue and cytokine secretion, shown to be modulated by circadian rhythms (62). Thus, their immune rhythms may also play an important role in β cell dysfunction. Chronic exposure to hyperglycemia causes oxidative stress which damages the organelles such as the mitochondria and endoplasmic reticula, leading to the apoptosis of these cells. Inflammation and oxidative stress contribute to the accumulation of reactive oxygen species in β cells, which hinders the mitochondrial electron transport chain and further damages organelles, thus exacerbating β cell dysfunction.

Although obesity and insulin resistance are risk factors for developing T2DM, not everyone who has these risk factors will progress to developing this condition (63). Indeed, some insulin resistant, obese individuals remain euglycemic because their β cells compensate by secreting more insulin. Genetic factors are determinants of whether or not β cell dysfunction develops in these individuals and there are several genetic variants which may protect or predispose to T2DM (64, 65).

Dietary factors are key risk factors for developing insulin resistance and β cell dysfunction (66). For example diets containing high amounts of saturated fats cause increased levels of circulating fatty acids, which is a risk factor for developing insulin resistance (67). Fatty acids compete with glucose for uptake and metabolism by tissues. Therefore, hyperglycemia will further increase free fatty acid concentrations in the blood, leading to a glucolipotoxic state which is toxic to β cells (59).

## Circadian rhythms in the pancreas

Blood glucose homeostasis and insulin resistance are strongly influenced by both local (i.e. pancreas) and distal (i.e. liver, intestine, brain) circadian rhythms, impacting multiple cell types and the secretion of many hormones (**Figures 4**, **5**).

It is clear that the secretion of insulin and glucagon, insulin sensitivity and glucose tolerance all display circadian rhythmicity (68–73), which can be disrupted in individuals with T2DM and their first degree relatives (74). Glucagon secretion is also controlled in a rhythmic manner; however, the circadian rhythms in both β and α cells are in different phases allowing them to respond accordingly to the local changes in glucose and insulin concentrations respectively (75). Bilateral thermic SCN ablation in rats has demonstrated the role of the central clock in glucose metabolism as in these rats, the diurnal patterns of glucose levels and insulin and glucagon secretion became arrythmic (70, 76). Furthermore, this SCN ablation caused desynchrony between peripheral clocks, indicating that the SCN master pacemaker maintains synchronization under normal physiological conditions (77, 78).

In addition to this, cortisol is secreted in a rhythmic, diurnal manner, with peak levels occurring shortly after waking in the morning (79, 80). Circadian disruption and misalignment are associated with aberrant cortisol secretion patterns (81), while a flattened diurnal cortisol curve and a diminished cortisol awakening response have both been associated with T2DM (82–84).

Both GIP and GLP-1 display circadian rhythmicity in humans and disruptions of these secretory patterns have been associated with obesity and T2DM (85–87). Furthermore, it has been postulated that GLP-1 is a key component of peripheral metabolic clocks, which entrains pancreatic, hepatic and gut clocks to daily patterns of nutrient intake (88, 89).

Ghrelin secretion oscillates in a circadian pattern which is reciprocally correlated to insulin secretion patterns (90). Immunolabelling studies show that ghrelin-responsive neurons in brain centers, including the ARC, receive direct synaptic input from the SCN, indicating that the downstream effects of ghrelin are regulated by the circadian timing system (91).

Thus, the regulation of blood glucose homeostasis is strongly influenced by circadian rhythms, both directly in the pancreas and through influences in other peripheral tissues i.e. the intestine, brain and liver. The successful coordination of these rhythms between the different tissues is paramount for maintaining good health. Preclinical animal model as well as human studies have been performed to investigate the role of the circadian molecular clock in regulating glucose homeostasis, insulin sensitivity and energy expenditure as discussed in more detail next.

## Molecular circadian rhythms in the pancreas

**Figure 6:**
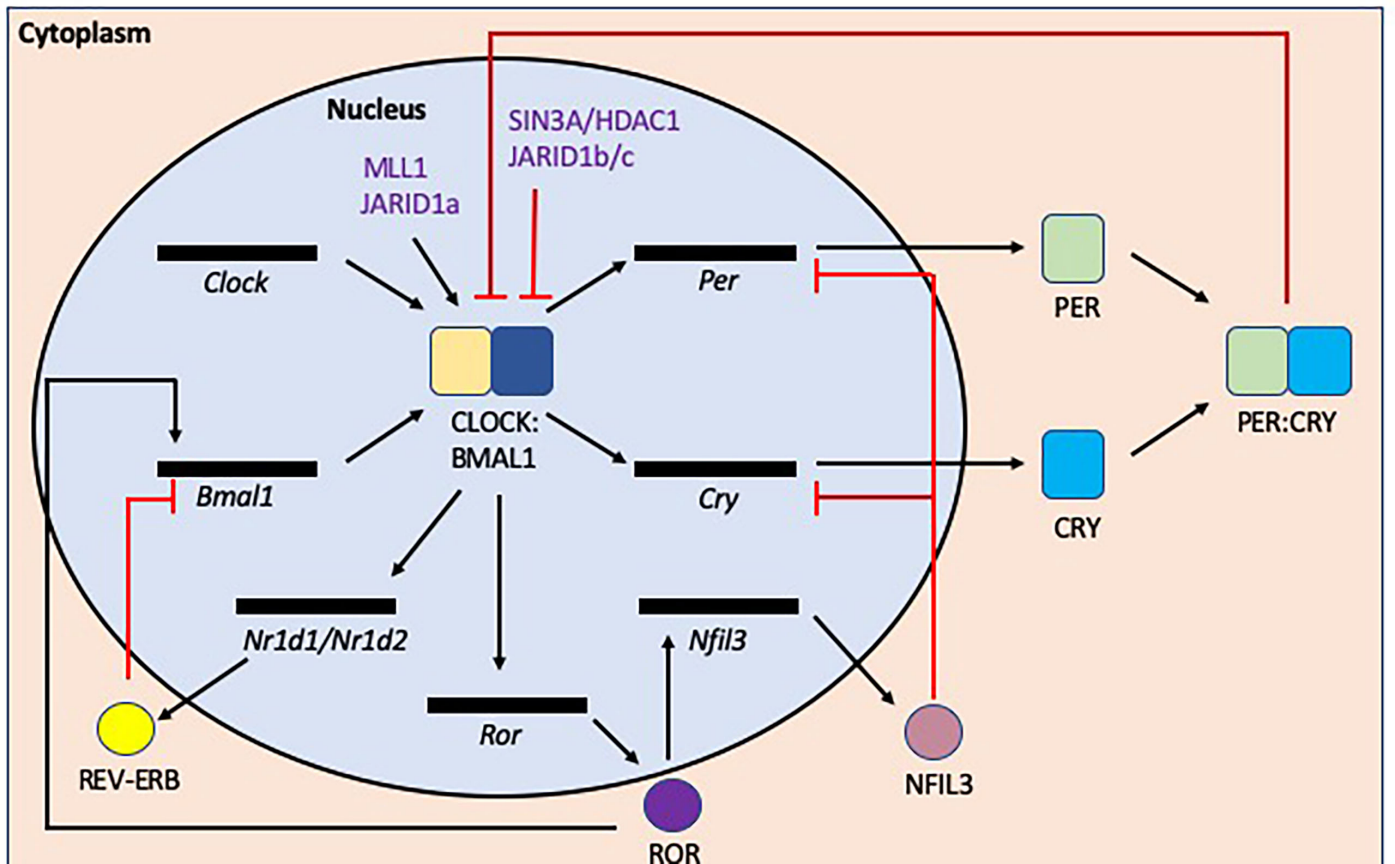
Transcription/translation feedback loops that modulate circadian rhythms at the molecular level. The induction of circadian rhythms relies on oscillations in gene expression and repression. In the initiation of the circadian rhythms, *Bmal1* and *Clock* are transcribed/translated and then form a heterodimer. This CLOCK : BMAL1 heterodimer initiates the transcription of a number of genes including *Per*, *Cry* and *Nr1d1/Nr1d2* (*rev-erbα/β*) genes, all which negatively repress the circadian initiators BMAL1 and/or CLOCK. In addition, CLOCK : BMAL1also activates *Ror* transcription, promoting transcription of *Bmal1*, while also inducing *Nfil3* transcription/translation, which inhibits *Per* and *Cry* gene transcription. There are also epigenetic modulation factors e.g. (de)acetylation or (de)methylation that also regulate the circadian rhythm as shown in purple.

Circadian rhythms are coordinated by tightly regulated central and peripheral clocks which respond to environmental and behavioral cues such as light, food intake and sleep-wake cycles (92). At the molecular level (**Figure 6**), Brain and Muscle Aryl hydrocarbon receptor nuclear translocator (*Bmal*) 1 and 2, Circadian Locomotor Output Cycles protein Kaput (*Clock*), Cryptochrome (*Cry*) 1 and 2, Period (*Per*) 1-3 genes regulate circadian rhythms *via* transcriptional-translational feedback loops (92). CLOCK and BMAL1 form heterodimers which bind to E-box sequences (CANNTG, where N is any nucleotide) to promote the transcription of *Per* and *Cry* genes. After translation, PER and CRY proteins form heterodimers in the cytoplasm and subsequently translocate into the nucleus to inhibit CLOCK : BMAL1 complexes from promoting further transcription. This cyclical regulation of transcription is achieved through modulating clock-specific and ubiquitous histone modifying factors. For example, CLOCK contains a histone-acetyltransferase (HAT) domain and also recruits histone 3 (H3) methyltransferase MLL1 and JARID1a, which inhibits histone deacetylase 1 (HDAC1) promoting CLOCK : BMAL1 activation (93–95), while PER1, recruits the SIN3A/HDAC1 complex which prevents CLOCK : BMAL1 complexes from binding to promoter regions (96).

CLOCK : BMAL1 complexes also promote the transcription of the nuclear receptors REV-ERBα/β and retinoic acid receptor-related orphan receptors (RORs) α/β. ROR proteins encourage the transcription of BMAL1 whereas REV-ERB proteins inhibit transcription (97, 98). These opposing factors compete for the ROR Response Element (RORE) binding sites (AGGTCA preceded by a 5 base pair A/T rich sequence). ROR also promotes the transcription of Nuclear factor, interleukin 3 regulated (*Nfil3*), which suppresses the transcription of *Per* and *Cry* genes (99) and more recently has also been shown to influence intestinal lipid uptake and obesity (100). Thus, the circadian clock genes modulate susceptibility to metabolic disease, as shown in both mouse and human genetic studies.

### Mouse circadian genetic studies

Studies of gene-deficient mice have identified that deletion, or mutation, of any core circadian gene can lead to altered glucose homeostasis or weight gain. In this section, we discuss the impact these genes individually have on metabolic circadian functions.

Studies on homozygous Clock mutant mice, characterized by lengthened circadian periods due to a deletion of exon 19 and amino acid 51 in the C-terminal activation domain of *clock*, show that circadian feedback loops have a considerable role in regulating glucose metabolism (101, 102). These mice demonstrate hyperphagia, dyslipidemia, hyperglycemia and hyperinsulinemia, all of which are associated with type 2 diabetes, obesity and metabolic syndrome. Interestingly, the *clock* mutation in these mice also reduces islet size and β cell proliferation (13), indicating an important role for circadian rhythms in both islet and β cell development. This suggests circadian rhythm modulation may promote β cell development and expansion, thus limiting metabolic dysfunction.

Mice deficient in *bmal1* displayed impaired adipogenesis and hepatic carbohydrate metabolism (11, 12). Global *bmal1* deficiency resulted in a blunted response to hypoglycemia, due to reduced hepatic gluconeogenesis, whereas liver-specific *bmal1*-deficiency resulted in impaired glucose tolerance (11, 103). Pancreas-specific *bmal1* gene knock out (KO) models develop hyperglycaemia and hypoinsulinemia, both of which are characteristic of diabetes development (13). Together, these suggest fundamental roles for intrinsic circadian responses in individual cell types. Understanding the roles of individual cell types in modulating these rhythms, and how that impacts on crosstalk with other cell types, will be fundamental in developing targeted therapeutic strategies to be explored.

*Per* and *cry* are downstream target genes of Clock/Bmal1. Homozygous *per2* knockdown mice had lower total triacylglycerol and non-esterified fatty acids compared with their wild-type (WT; i.e. *per2*-sufficient) counterparts; however there was no difference in the expression of other clock genes in white adipose tissue (WAT) (104). In this study, Grimaldi and colleagues found that Per2 is likely to regulate lipid metabolism through a PPARγ2-dependent mechanism (104). Per3 is also involved in lipid metabolism and *Per3*-deficient mice are more prone to weight gain, when exposed to a high-fat diet (HFD), compared to their WT counterparts (105).

Murine *cry1* and *cry2* gene deletions are associated with disruptions in the circadian rhythmicity of insulin and glucagon secretion, which in turn are associated with insulin resistance, hypertension and impaired glucose tolerance and dyslipidemia (106, 107). Similarly, agonists of Cry1 and Cry2 inhibit hepatic gluconeogenesis *in vitro (* 108). In contrast, liver-specific expression of adenoviral encoded *cry1*, at a time when Cry1 is endogenously low, appeared to protect the mice from these metabolic risk factors and increased insulin sensitivity, whilst decreasing blood glucose (109). These differences may relate to cell-specific roles, differences in mice studied (food-restricted or not), timing and methodology of alteration (i.e. lifelong gene deficiency or induced expression following adenovirus delivery) or alterations in other circadian gene regulation or expression.

Rev-erbα plays an important role in regulating insulin and glucagon secretion and pancreatic β cell proliferation (110). Downregulation of *rev-erbα* using siRNA in mouse pancreatic islet cells reduces glucose-stimulated insulin secretion (GSIS) in mouse models (111). The authors of this study found that exogenous leptin treatment enhanced *rev-erbα* expression *in vitro* and *in vivo*, whilst a HFD further downregulated *rev-erbα* expression. Mouse models lacking *rorα* and *rev-erbα* have lower high-density lipoprotein and decreased adiposity compared to their WT counterparts (97, 112). Solt and colleagues showed that Rev-erb agonists can decrease fat mass and total serum cholesterol in diet-induced obese mice (113). Similarly, Rorα inverse agonists are effective at preventing hyperglycaemia in mouse models of type 2 diabetes (114).

Thus, studies of mice have greatly helped to identify key mechanisms and cell-specific contributions that aid in the modulation of circadian rhythms, leading to altered susceptibility to obesity and diabetes development. It is clear that this is a growing field and more understanding is required of how cell intrinsic clocks impact on other cell types and how other environmental factors may alter peripheral oscillations, leading to altered susceptibility to obesity and diabetes development. Further knowledge of how we can modulate the circadian rhythms in humans will be vital, but animal models may be very helpful for developing preclinical therapies for translation into humans.

### Human genetic studies

There are single nucleotide polymorphisms (SNPs) in humans that have been associated with the risk of developing metabolic dysbiosis, obesity and T2DM (**Table 1**). *Clock* SNPs can predispose individuals to developing obesity, metabolic syndrome and T2DM by altering the metabolism of fatty acids, as well as the monosaturated fatty acid content of red blood cells (115–118). Similarly, a SNP in NPAS2, a paralog of CLOCK, which can also bind to BMAL1 (126), has also been linked to risk factors (e.g. hypertension) for developing metabolic syndrome (119). SNPs in *Bmal1* have also been associated with hypertension, hyperglycemia, T2DM and gestational diabetes (120, 121). In addition, SNPs in CLOCK : BMAL1-repressing genes, such as *Cry* and *Per* genes, have also been implicated in metabolic disease. Both *Cry2* and *Per2* SNPs have been associated with impaired glucose tolerance (119, 122), while *Per2* SNPs have also been associated with binge eating and stress related to dieting, leading to increased weight gain (123). These core circadian rhythm-inducing genes, modulate the rhythmic expression of many other genes in the body. One example is the rhythmic secretion of melatonin, which in humans increases in the evening and decreases in the daytime, aiding in regulating our sleep/wake cycles (127). Interestingly, two SNPs in one of the melatonin receptors, the melatonin receptor 1B gene (*Mtnr1b*), have been associated with higher fasting glucose concentrations, reduced β cell function (as measured by homeostasis model assessment (HOMA)) and an increased risk of developing T2DM (124, 125). This SNP appears to influence the dynamics of melatonin secretion, which may modulate the susceptibility to developing T2DM (128). This suggests important roles for both the SNPs involved in the molecular circadian clock, but also their downstream genes in modulating susceptibility to metabolic syndrome, obesity and T2DM.

**Table 1:** SNPs in circadian rhythm-related genes associated with metabolic dysfunction, obesity and T2DM in humans.

Gene and location	SNP [allele(s)]	Study Population	Association	Reference
*Clock* (4q12)	rs1554483 (G) rs4580704 (C) rs68437222 (C) rs6850524 (G) rs4864548 (A) rs1554483-rs4864548 (GA)	Lean (n=715) and overweight/obese (n=391) individuals of self-reported European descent in Buenos Aires, Argentina.	Up to 1.8-fold risk of developing overweight/obesity	(115)
	rs4580704 (CC) rs1801260 (C)	1100 American individuals of European descent	Increased risk of developing metabolic syndrome components	(116)
	rs4864548-rs3736544-rs1801260 (CAT)	537 individuals from 89 British families (all white European)	Associated with presence of metabolic syndrome	(117)
	rs4580704 (CC)	7098 individuals with T2DM or with 3 or more cardiovascular risk factors (all European)	Increased fasting glucose, and increased development of T2DM. Increased risk of cardiovascular disease in individuals with T2DM.	(118)
*Npas2* (2q11)	rs11541353 (C)	517 Finnish individuals	Associated with hypertension	(119)
*Bmal1 (*11p15)	rs7950226-rs11022775 (haplotype AC)	1304 individuals from 424 British families with T2DM of European descent	T2DM	(120)
	rs6486121-rs3789327- rs969485 (CCA)		Hypertension	
	rs7950226 (A) rs11022775 (C) rs7950226-rs11022775 (GC) rs7950226-rs11022775 (AC)	185 women with Gestational diabetes and 161 controls (Greek population)	Increased risk of developing gestational diabetes	(121)
*Cry2* (11p11)	rs11605924 (A)	21 GWAS cohorts including up to 46,186 non-diabetic individuals, with a further follow up of 25 loci in 76,558 additional individuals of white European descent from United States or Europe	Higher fasting glucose levels	(122)
*Per2* (2q37)	10870 (A)	517 Finnish individuals	Increased risk of raised plasma glucose	(119)
	rs2304672 (G) rs4663302 (T)	454 overweight/obese Spanish individuals	Increased snacking, higher stress when dieting, more likely to eat when bored Higher waist circumference and waist to hip ratio	(123)
*Mtnr1b* (11q14)	rs1387153 (T)	2151 non-diabetic (encompassing lean and obese) French subjects with European ancestry. Replication analysis conducted in 5,518 middle-aged non-diabetic Danish individuals, 3,886 and 1,453 non-diabetic French individuals from 2 cohorts and 5,237 young (16 years of age) Finnish individuals	Increased fasting blood glucose, increased risk of developing hyperglycemia and T2DM	(124)
	rs10830963 (G)	10 GWAS study cohorts and 13 case-control studies (18,236 cases, 64,453 controls) of European descent	Increased fasting blood glucose levels, reduced beta cell functions and an increased risk of developing T2DM	(125)

Information on the expression of clock genes in human pancreatic islets is limited, but circadian genes are expressed in human islets (129). In individuals with T2DM, *Cry2*, *Per2* and *Per3* expression was reduced in the islets compared to islet donors without T2DM (130). Additionally, *in vitro*, islets cultured in glucolipotoxic conditions (16.7mmol/L glucose per 1mmol/L palmitate) for 48 hours downregulated the expression of *Per3* in the pancreatic islets of individuals without T2DM (130). The aforementioned studies highlight the importance of circadian clock genes in regulating metabolic functions such as glucose tolerance and β cell function; however, many of these studies did not investigate the expression of these genes at the protein level. Although Stamenkovic and colleagues correlated mRNA expression to corresponding protein concentrations in human islets, post-transcriptional factors such as miRNA and post-translational modifications were not examined in this study (130). Future studies that address these interactions and mechanisms of regulation may provide additional insights into the relationship between the circadian clock and metabolic physiology. Additional studies, particularly in non-white European populations, with increased numbers of participants are also greatly needed.

## Modulation of circadian rhythm

**Figure 7:**
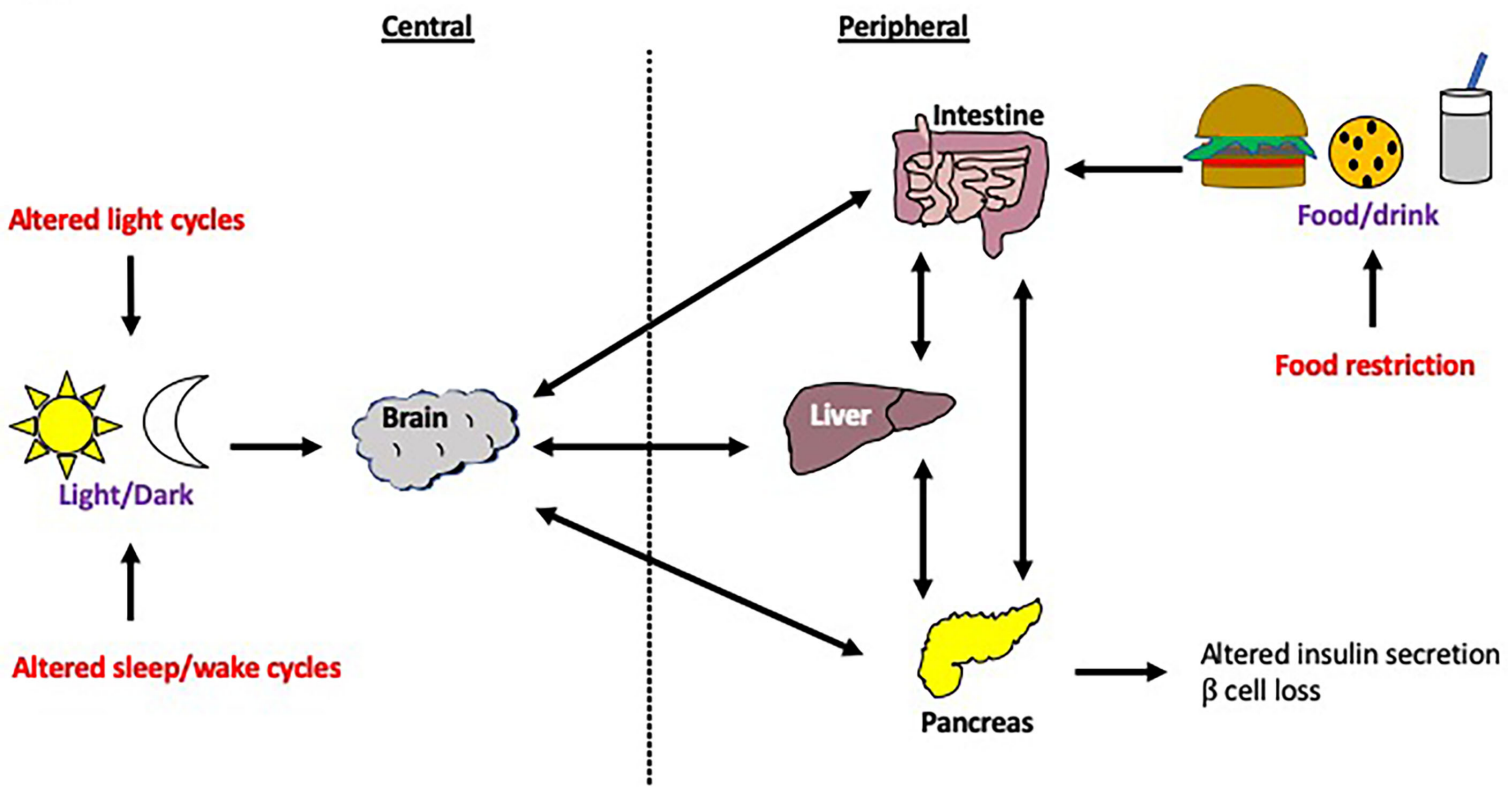
Modulation of host circadian rhythms. Circadian rhythms can be modulated through a number of interventions, including changes to the central clock *via* changes in the light/dark cycle or sleep/wake cycle, as well as changes to peripheral clocks e.g. food restriction. Disruption to the central clock can lead to disconnection from the peripheral clocks and vice versa. These changes to the circadian rhythm are often experienced by those with jet lag or in overnight shift workers. .

Misalignment between peripheral and central clocks is associated with insulin resistance, metabolic abnormalities and cardiovascular disease (17, 131, 132). This desynchrony can be achieved experimentally through a forced desynchronization (FD) protocol which involves altering behavioral patterns, such as feeding and sleep-wake cycles, so that they are substantially longer or shorter than 24 hours, whilst ensuring that the subjects are only exposed to dim light during their wake times (133). The aim of this is to desynchronize endogenous circadian rhythms from external influences e.g. food intake, light exposure as outlined in **Figure 7**. Buxton and colleagues demonstrated that a FD protocol (sleep restriction and circadian disruption) increased plasma glucose levels in human studies (134). Although the mechanism for this is unclear, a study which utilized human islet amyloid polypeptide (HIP) transgenic rats showed that circadian disruption accelerated the β cell loss and dysfunction in this model of T2DM (135). Furthermore, sleep deprivation studies demonstrated disrupted rhythmicity of insulin and glucagon levels, as well as insulin sensitivity and glucose tolerance (136–138). In these studies, circadian rhythm cycles have clearly influenced susceptibility to metabolic disease. In this section, we break down the different environmental cues that can significantly alter circadian rhythms in animal models and humans.

### Altered light cycles

In mice, electrophysiological monitoring has shown that exposure to constant light reduced the amplitude (the difference between peak and trough) of SCN rhythmicity (139). This resulted in increased food intake, whilst energy expenditure was decreased. These mice also showed a complete loss of rhythmicity in insulin sensitivity, energy expenditure and food intake. In humans, light intensity has been shown to regulate postprandial glucose levels and triglycerides (140). In this study, healthy lean men and obese men with T2DM were exposed in the morning to 5 hours of either bright light (4000 lux) or dim light (10 lux), with a 600kcal meal given 1 hour after the start of the light exposure. While no changes were seen in the fasting or postprandial glucose levels of healthy lean men between dim or bright light exposure, obese men with T2DM had improved fasting and postprandial glucose levels, when exposed to bright light. In addition, healthy lean men exposed to bright light had higher fasting and postprandial plasma triglycerides, while in obese men with T2DM bright light only increased postprandial plasma triglycerides and did not change fasting triglyceride concentrations. Similar results were also seen in a study of individuals with insulin-resistance exposed to either bright day – dim evening light or dim day – bright evening light conditions (141). Thus, the light exposure can significantly impact our glucose homeostasis.

### Altered diets and time of food intake

A high-fat diet also disrupts central and peripheral clocks in mouse models, including hypothalamic, adipose and hepatic clocks (142). The timing of food intake also affects circadian clocks. When kept in a 12-hour light/12-hour dark cycle (12:12h LD), mice consume the majority of their food during the dark phase when food is available *ad libitum (* 143). In contrast, during timed food restriction, mice fed a HFD only during the light phase gained more weight than those fed during the dark phase (144). Similarly, mice fed a HFD restricted to the dark phase are less likely to develop metabolic abnormalities such as obesity and glucose intolerance than mice fed the same HFD *ab libitum (* 145). Time-restricted feeding, where food is provided for a specific duration only, is also effective at preventing obesity and metabolic syndrome in circadian gene-deficient mice (whole body *cry1/2*-deficient mice and liver-specific *bmal1* and *rev-erbα/β-*deficient mice*)* (146). Feeding restricted to the light phase also caused a desynchrony of peripheral clocks in the pancreas, liver, heart and kidneys by up to 12 hours, which did not affect the SCN (147). Mice exposed to calorific restriction in 12:12h LD cycles will become partly diurnal, as opposed to purely nocturnal and this is attributed to changes in peripheral clocks and the SCN (148, 149).

As in the animal studies, the timing of eating also influences risk of diabetes in humans. For example, a randomized crossover study showed that a later dinner was associated with impaired glucose tolerance in a subset of MTNR1B (melatonin receptor 1B) risk allele carriers (150). The postulated role of melatonin in this process agreed with a previous study showing that exogenous melatonin could also cause impaired glucose tolerance (151). Conversely, feeding restricted to 9 hours improved glycemic control in men with type 2 diabetes (152). Similarly, time-restricted eating (TRE) also improved metabolic parameters such as weight, visceral fat, atherogenic lipids and blood pressure in individuals with metabolic syndrome (153, 154); however, these benefits of TRE were observed in small sample sizes (n=15-20) and are currently under investigation larger cohorts (155, 156). Maintaining TRE after weight is potentially a challenge and further research would also be necessary.

In a FD protocol study involving 5 male and 5 female adults, increased blood glucose levels were coupled with a paradoxical rise in insulin secretion during the misalignment phase (where eating/sleeping is 12 hours out of synchrony with the normal schedule) (131). Another study which combined a FD protocol, with restricted sleeping hours (6.5 hours in a 28-hour day), showed a similar rise in glucose levels in the individuals, coupled with increased insulin secretion (134). A possible explanation for this may be reduced insulin sensitivity secondary to circadian misalignment. This is supported by a 12-hour rapid shift work protocol, which utilized the hyperinsulinemic-euglycemic clamp and showed that circadian misalignment is associated with decreased insulin sensitivity (157).

The timing of nutrient intake also alters the circadian rhythmicity of the gut bacterial composition in mice (158, 159). For example, bacterial species belonging to the phylum Firmicutes thrive postprandially in response to dietary glycan intake, whilst the phyla Bacteroidetes and Verrucomicrobia usually peak in numbers during fasting periods (158–160). As mentioned, the gut microbiota can oscillate, altering important metabolic functions in mice. In line with this, a recent study in humans identified a gut bacterial signature, encompassing 13 taxa with disrupted rhythmicity, which, in conjunction with BMI, could be used to predict individuals who would later develop T2DM (161). Thus, host-microbial rhythms may act as a biomarker for disease development. Interestingly, common gastric bypass procedures such as Roux-en-Y gastric bypass, which enable individuals to lose weight, are associated with altered microbial composition (162). It would be interesting to assess whether these procedures also alter host and microbial rhythmicity.

### Shift work

As mentioned previously, circadian misalignment, and by extension shift-work, is a risk factor for developing metabolic syndrome, obesity and type 2 diabetes (18). Circadian misalignment as a risk factor for metabolic abnormalities has been corroborated in a real-life study that compared day-shift and night-shift workers (16). In this study, night-shift workers were found to have increased postprandial glucose and insulin levels as well as elevated triacylglyerol levels, compared to day-shift workers. A meta-analysis of 12 observational studies revealed that shift-work is associated with a 9% increase in the chance of developing T2DM compared to people who have not been exposed to shift work (19). Importantly, people who have rotating shift work are more at risk than those employed in constant shift work (22). This is likely due to exposure to both light and food intake at times-of-day different to when the body naturally anticipates these changes. Furthermore, a rat study using a simulated shift-work protocol (using rotating running wheels) was consistent with the human data and caused the animals to develop abdominal obesity and blunted rhythmicity of glucose levels (163). In rats, disruption of 12:12h LD cycles, with continuous light exposure, accelerated β cell dysfunction and loss, whilst impairing GSIS (135, 164). Studies on β cell-specific *bmal1* knockout mice showed that Bmal1 plays an important role in adapting to circadian disruptions and preventing oxidative stress (14). Thus, a loss of function of this gene predisposes mice to β cell dysfunction and further to diabetes.

### Jet lag

Jet lag is also associated with circadian misalignment and is characterized by a series of psychological and physiological symptoms such as low mood, impaired cognitive performance, loss of appetite, gastrointestinal disturbances and general malaise (165). Chronic jet lag may elevate the risk of developing cancer, cardiomyopathy and T2DM (166–168) and can be simulated in animal experiments by altering the duration of light or dark cycles (169). Studies utilizing this approach have found that there is extensive desynchrony between different body tissues and also variation in the time taken by different tissues to adjust to new light/dark cycles (170). Adrenal glucocorticoids (GCs) appear to play a key role in the re-entrainment process of circadian rhythms in jet lagged mice (171–173). Injection of metyrapone (MET; an inhibitor of corticosterone synthesis) prior to performing the jet lag protocol, was found to prolong re-entrainment when administered in the inactive phase and accelerate re-entrainment when given during the active phase (170). Since the SCN does not express GC receptors, GCs are unlikely to directly feedback to the SCN to regulate this process (173, 174). It has been hypothesized that dysregulation of the adrenal clock may cause aberrant secretion of other adrenal hormones such as aldosterone alongside GCs, which may feedback to the master pacemaker to regulate re-entrainment (175).

In jet-lagged mice, both the composition of the bacteria and metabolic functions were altered compared to control mice (158). These mice experienced impaired glucose tolerance and obesity caused by the bacteria as (a) these metabolic abnormalities were diminished following the eradication of the microbiota using antibiotics and (b) the transplantation of microbiota from these jet-lagged mice to germ-free mice, which lack all bacteria, transferred these metabolic abnormalities to the recipient mice. Together, these studies suggest that there is a functional relationship between host and bacterial rhythms which are important in modulating metabolic dysbiosis. Thus, alterations of the bacterial composition or oscillations could potentially be harnessed to prevent obesity, metabolic abnormalities or T2DM.

Social jet lag, defined as a temporal discrepancy between a person’s sleep pattern on working days and non-working days (23), has also been associated with diabetes susceptibility. Individuals with greater than 1 hour of social jet lag have a 75% greater chance of developing diabetes or prediabetes, compared to people with less than 1 hour of social jet lag (21).

Together, rodent and human studies have identified that the timing of food intake, nutrient content and light exposure are important stimuli in regulating the metabolic clock. These findings indicate that novel interventions such as time-specific therapy (chronotherapy) or interventions which target the circadian system such as synthetic circadian protein analogues may be beneficial in the future management of metabolic syndrome and T2DM (176).

## Chronotherapy

Chronotherapy is the concept of administering drugs or other treatments at optimal times of the day in order to produce the most benefit (177). In clinical practice, statins are typically taken in the evening as their mechanism of action is to inhibit 3-hydroxy-3-methylglutaryl coenzyme A (HMG CoA) reductase, an enzyme which peaks in concentration in the night (178). There are many other examples of chronotherapy already in existence and its importance in relation to diabetes management as discussed below.

Bromocriptine is a dopamine agonist which is used as an adjunct in the treatment of T2DM (179). Dopaminergic activity in the hypothalamus follows a circadian rhythm and drives hepatic gluconeogenesis and adipocyte lipolysis. Bromocriptine is thought to reduce this drive when given within 2 hours of waking to prevent hyperglycaemia and dyslipidaemia. Although bromocriptine demonstrated efficacy in glycemic control and has been approved by the Food and Drug administration (FDA) in T2DM, this drug is infrequently used in clinical practice (52, 180, 181).

Despite preclinical studies showing that metformin interacts with molecular components of the circadian system and has time-dependent effects on blood glucose (182–184), there have not been any clinical studies to investigate the timing of metformin administration. This may be especially important given oscillations in both host and bacterial rhythms, as metformin alters the bacterial composition and function to mediate therapeutic effects of the drug (185). As metformin is a first line therapeutic for T2DM, it would seem particularly important to maximise efficacy. Similarly, the short-acting sulfonylurea drug tolbutamide appears to have time-dependent effects on insulin secretion but its chronotherapeutic potential is yet to be investigated (186).

Targeting circadian rhythm proteins may also be important for preventing/reversing metabolic dysbiosis. REV-ERBα agonist SR9011 and REV-ERBβ agonist SR9009 reduced obesity and hyperglycaemia in mouse models (113). Both of these synthetic REV-ERB agonists induced increased energy expenditure in white adipose tissue. Furthermore, the naturally-derived compound nobiletin activates circadian molecules RORα and RORγ and prevents metabolic syndrome from developing in diet-induced obese mice (187). Synthetic CRY stabilizers have also been reported to have a protective effect against diabetes by improving glucose tolerance in mice (188). In these rodent studies, these drugs have a short half-life and are typically administered by injection at intervals shorter than 3 hours to maintain suitable bioavailability (189). It has been hypothesized that humans would eliminate the active metabolites of these drugs even more quickly, therefore requiring more frequent injections (189). In a clinical context, this may not be practical. Therefore, this pharmacological obstacle must first be addressed before studies in humans can be carried out effectively. Novel therapies for T2DM, obesity and metabolic syndrome may be identified if these and similar drugs can demonstrate similar effects in human studies.

## Future directions

In today’s industrialized world, only a minority of people have an internal sleep-wake cycle which is consistent with their social commitments (190). Therefore, it is unsurprising that social jetlag is common in the population (23). An individual’s personal circumstances has a substantial influence on many of the exogenous factors which influence circadian rhythms including sleep-wake cycles, exposure to light, eating times and activity level (191). These environmental factors, together with endogenous characteristics such as age, genetics and chronotype (i.e. the time of day people are most alert/sleep), influence the degree of circadian desynchrony which an individual experiences (9).

In clinical practice, there is currently neither a standardized scoring system which precisely encompasses the exogenous determinants of circadian activity, nor is there a genetic screening program which identifies carriers of genetic variations predisposing people to circadian disruption. The future development of these risk stratification tools may influence clinical practice by allowing disease management to be tailored to an individual’s circadian rhythmicity. For example, the timing of drug administration could be regulated in order to maximize efficacy, which may enable reduced drug concentrations to be used, thus limiting any toxicity or potential side effects. In order for this to be achieved, further epidemiological studies are necessary to quantify the relative risk of circadian disruption associated with different behavioral and genetic risk factors.

There are many exogenous factors which can alter host/microbial rhythmicity, which can then modulate susceptibility to metabolic dysfunction and T2DM. Recent evidence has shown arrhythmic bacterial signatures could be used as a biomarker to predict individuals who would later develop T2DM; thus, further investigation into host/microbial rhythms, or lack thereof, as biomarkers for predicting metabolic dysfunction or the onset of diabetes should be conducted. Non-pharmacological interventions can also be used to target the circadian system in metabolic disease. Currently, trials investigating the effects of time-restricted feeding are taking place on large cohorts with type 2 diabetes and metabolic syndrome (155, 156). The results from these studies will provide insight into the potential for simple lifestyle changes that can modulate circadian rhythms as a therapy. Understanding the mechanism behind these changes will be vital.

## Summary

Human epidemiological and genetic studies have highlighted the importance of circadian rhythms in metabolic diseases such as T2DM and metabolic syndrome. This has led to extensive research in animal models and humans, which have concluded that circadian dysregulation and misalignment is associated with the development of metabolic abnormalities. Clinical applications of this knowledge may include the optimization of existing antidiabetic therapies such as metformin. Circadian molecules such as nobiletin, REV-ERB agonists and CRY stabilizers have demonstrated efficacy in preclinical studies and may lead to the development of novel treatments for diseases linked to circadian dysregulation. However, limitations in current knowledge mean that further research is required before these interventions can be used clinically.

## Author contributions

KC and JP wrote the manuscript. FW and JP edited the manuscript. All authors contributed to the article and approved the submitted version.

## Funding

This work was funded by a Medical Research Council Career Development Award (MR/T010525/1) and a JDRF UK small grant award (1-SGA-2021-0002) to JP and a Medical Research Council research grant (MR/K021141/1) to FW.

## Conflict of interest

The authors declare that the research was conducted in the absence of any commercial or financial relationships that could be construed as a potential conflict of interest.

## Publisher’s note

All claims expressed in this article are solely those of the authors and do not necessarily represent those of their affiliated organizations, or those of the publisher, the editors and the reviewers. Any product that may be evaluated in this article, or claim that may be made by its manufacturer, is not guaranteed or endorsed by the publisher.
